# The SNPs in *myoD* gene from normal muscle developing individuals have no effect on muscle mass

**DOI:** 10.1186/s12863-019-0772-6

**Published:** 2019-09-02

**Authors:** Suying Ding, Yaping Nie, Xumeng Zhang, Xiaohong Liu, Chen Wang, Renqiang Yuan, Keren Chen, Qi Zhu, Shufang Cai, Ying Fang, Yaosheng Chen, Delin Mo

**Affiliations:** State Key Laboratory of Biocontrol, School of Life Sciences, Sun Yat-sen University, Guangzhou, 510006 Guangdong China

**Keywords:** Muscle mass, Myogenesis, Nucleotide variation, Pig, Transcriptional activity

## Abstract

**Background:**

Myogenic Differentiation 1 (MyoD) is a crucial master switch in regulating muscle-specific gene transcription. Forced expression of *myoD* is equipped to induce several cell lineages into myoblast, which then differentiate and fuse into myotube. Pig is one of the most significant livestock supplying meat, and has been classified into lean, fat and miniature pig breeds. However, the mechanisms underlying muscle mass variation among different pig breeds have remained unclear. Considering the important effect of MyoD on muscle development, it remains to be investigated whether the difference in muscle mass is caused by its single nucleotide polymorphisms (SNPs) which are the major differences among pig breeds at DNA level.

**Results:**

In this study, we identified the locations of porcine *myoD* regulatory regions including proximal regulatory region (PRR), distal regulatory region (DRR), and core enhancer (CE) region. There are 8 SNPs in the regulatory regions and 6 SNPs in gene body region, which were identified from lean, fat and miniature pig populations. However, these SNPs have no effects on its temporal expression and transcriptional activity which might lead to the distinction in postnatal muscle mass. In addition, overexpression of *myoD* clones across from amphibious to mammals including xenopus tropicalis, chicken, mouse and pig whose gene identities vary from 68 to 84%, could promote myogenesis in NIH3T3 fibroblasts cells.

**Conclusions:**

These results proved that *myoD* nucleotide variations from different pig populations have no effect on muscle mass, suggesting that the function of *myoD* is highly conserved not only among different pig breeds, but also across different species. Thus, it would be futile to discover SNPs affecting muscle mass in pig populations with normal muscle development.

**Electronic supplementary material:**

The online version of this article (10.1186/s12863-019-0772-6) contains supplementary material, which is available to authorized users.

## Background

Different pig breeds vary in muscle mass because of genetic differences and intensive selection. Development of skeletal muscle determines muscle mass. Numerous researches on mechanisms of skeletal muscle development have been reported, and many genes regulating myogenesis have been found [1–3]. Among them, MyoD, containing basic helix-loop-helix (bHLH) domain, can bind to muscle-specific genes and promote their expression robustly [4, 5]. Previous study revealed that earlier *myoD* expression exhibits precocious myogenic differentiation and causes severe muscle hypotrophy [6]. In addition, our earlier research found that *myoD* showed differential expression at 35 days-post-coitus in Landrace and Lantang pigs [7]. These suggest the expression time and level of MyoD have an important effect on muscle mass. However, there are many factors affecting *myoD* expression, the primary one is its regulatory elements. So far, three regulatory regions have been identified to regulate *myoD* expression: proximal regulatory region (PRR), distal regulatory region (DRR), and core enhancer (CE) region. The CE region and DRR are essential to regulate *myoD* expression [8, 9]. Recent findings have found that *myoD* transcripts corresponding to CE and DRR enhancers can promote chromatin accessibility and RNA polymerase II recruitment at *myoD* and *Myogenin* loci, respectively [10–12]. In this case, is the difference in muscle mass among different pig breeds related to the SNPs in *myoD* gene? Therefore, we obtained *myoD* SNPs in its regulatory regions and gene body by resequencing, and analyzed them to explore whether these SNPs caused alteration in muscle mass in pigs. This study not only helps us to better understand the mechanisms of different muscle mass among pigs, but also provides new clues to study these mechanisms in the future.

## Results

### Determination of *myoD* regulatory regions in pigs

**Fig. 1:**
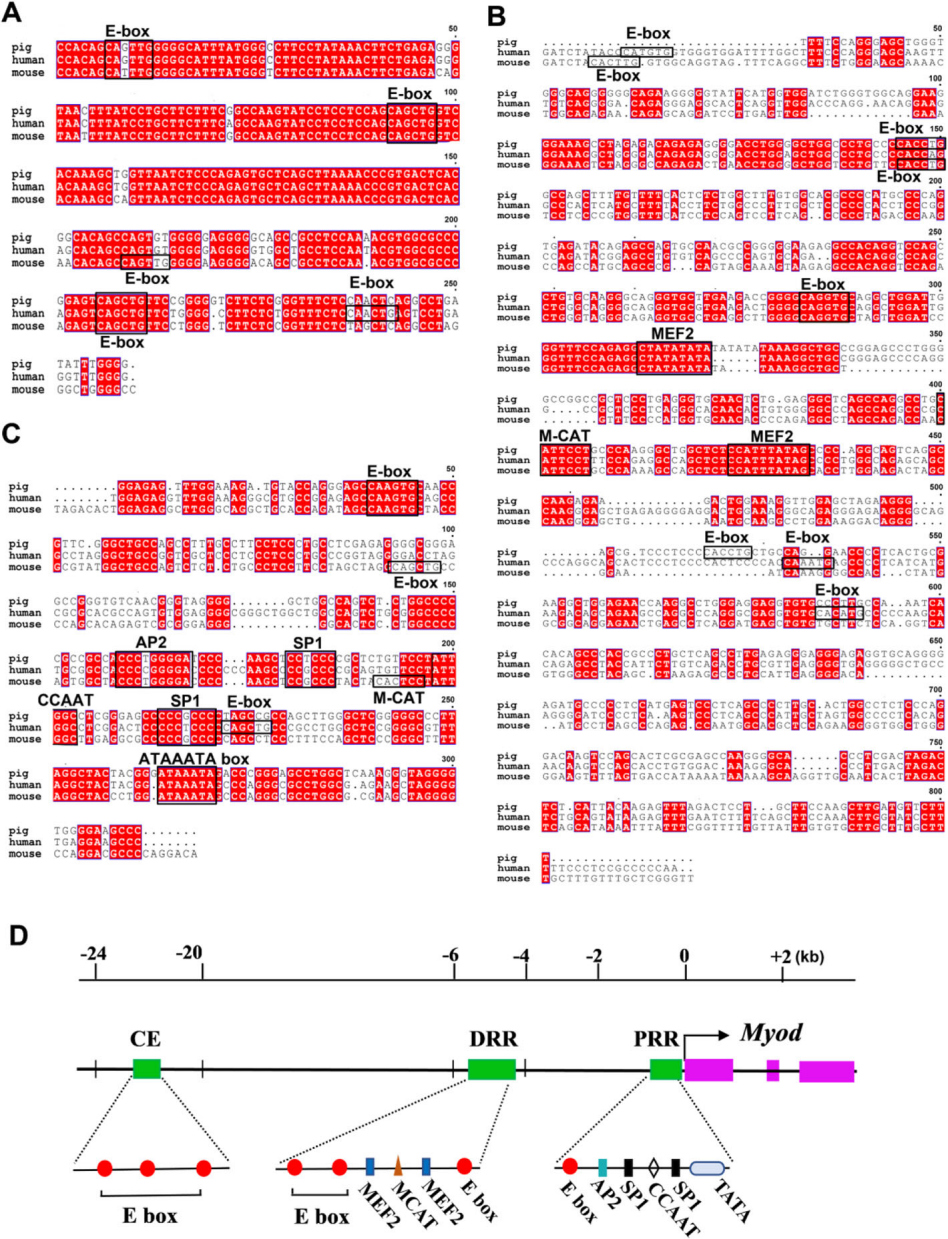
Sequence and location determination of *myoD* in pigs. (**a-c**) Homology of CE regions (**a**), DRR (**b**) and PRR (**c**) of humans, mice and pigs *myoD* were obtained respectively. The CE region is highly conservative among the three species. Regions of identity of three species are represented in red. Consensus or near-consensus binding motifs for factors implicated in muscle gene regulation (black boxes) were identified. **d** A statistical map of the regulatory region of *myoD* gene in pigs

The location of *myoD* regulatory regions in pigs have not been identified before. Depending on the CE, DRR and PRR of human and mouse *myoD* reported in documents [13–15], we finally identified the locations of porcine *myoD* CE regions (Fig. 1a), DRR (Fig. 1b) and PRR (Fig. 1c) by comparing nucleotide sequence similarities using BLAST. The CE region of porcine *myoD* was localized to a 258 bp fragment approximately 21,924 to 22,182 bp upstream of *myoD* transcriptional start site (TSS), which shows 94% sequence homology with human (Additional file 3: Table S3, Additional file 4: Table S4.). The DRR was located at 5 kb upstream relative to the TSS and the PRR was located between -197 bp and -485 bp (Additional file 4: Table S4).

In these regions, it was found that the CE region contains three E-boxes (Fig. 1a, d). In addition, the DRR contains consensus sequences for three E-boxes, two MEF2 binding sites and so on (Fig. 1b, d), which are nearly identical to human and mice [13, 15, 16]. The PRR contains consensus sequences for an E-box, two SP1 sites, an AP2 binding sites, etc. (Fig. 1c, d), which are necessary elements for mouse PRR to regulate muscle-specific transcription [15, 16].

### The SNPs in regulatory regions of *myoD* gene have no effect on muscle mass

Considering the significant role of *myoD* regulatory region in itself expression, we hypothesized that the polymorphisms of *myoD* are associated with its expression time and expression level resulting in the divergence of pig muscle mass. To find out whether the SNPs in *myoD* gene affect muscle mass, whole *myoD* genome sequence including regulatory regions and gene body were obtained from resequencing results. One SNP existed in all breeds in the CE region (C41446175T) listed in Table 1. However, this SNP also existed within the pig breed. Therefore, this SNP was unlikely to be the major cause that leads to the different muscle mass. Another SNP at locus 41,446,192 in the CE region was found only in individuals, but no breed specificity. The same results were also observed in DRR and PRR (Table 1). Moreover, these SNPs in the three regulatory regions did not appear in their binding motifs which were marked in black boxes (Fig. 1a, b and c). Therefore, these results indicate that SNPs in *myoD* regulatory region obtained from normally developed pig populations do not contribute to muscle mass.

**Table 1:**
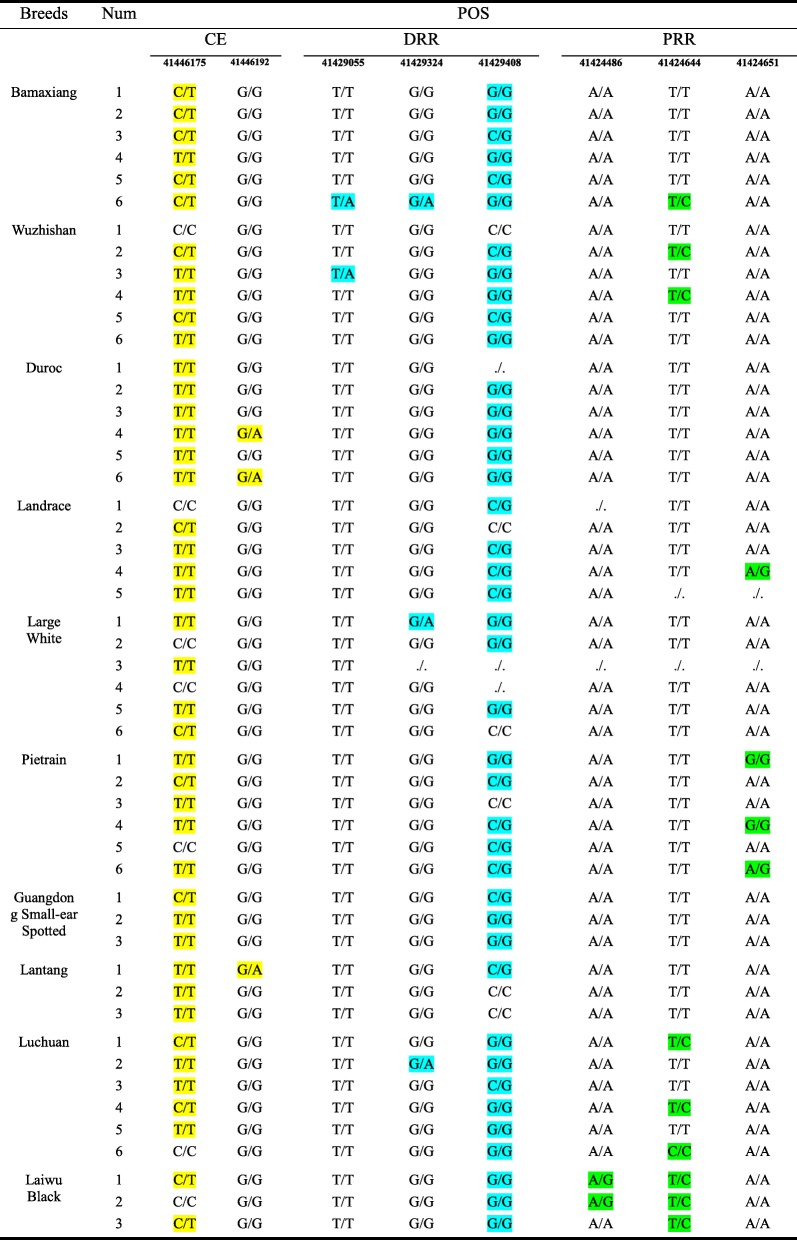
The SNPs in *myoD* regulatory region

(1) The SNPs in regulatory regions of *myoD* are shown and variations in each region are represented by the colors indicated in the table

(2) “./.” represents that the site has not been detected

### The mutation (Arg76Pro) has no effect on muscle mass

**Fig. 2:**
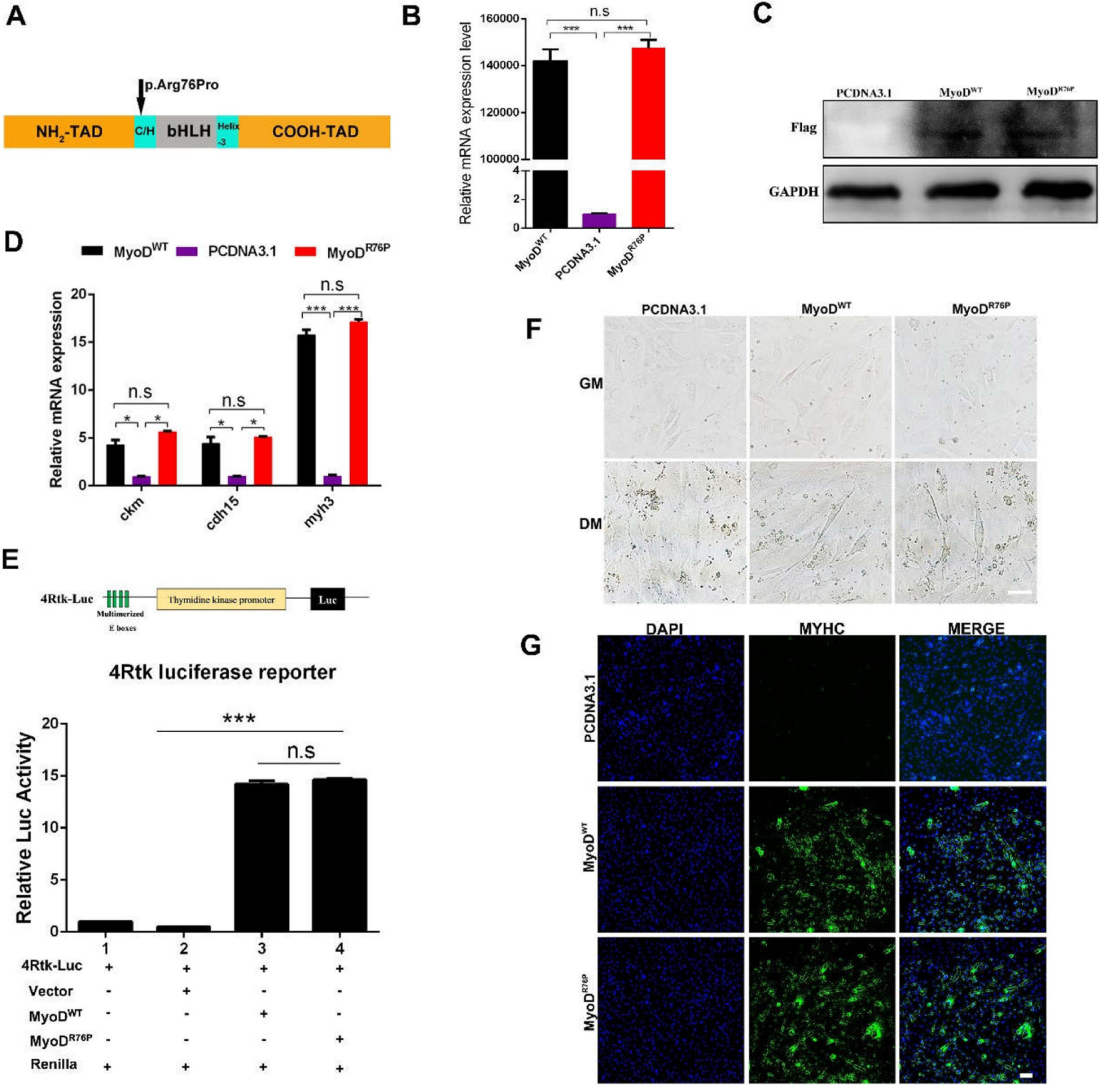
The mutation (Arg76Pro) of MyoD has no effect on its transcriptional activity. **a** Schematic representation of WT pig MyoD protein. Arrows denote amino acid positions of the mutation. **b** qPCR analysis for the expression of *myoD* in NIH3T3 cells treated with overexpression Flag-*MyoD*^*WT*^、Flag-*MyoD*^*R76P*^ or a control Flag vector. *GAPDH* was used as internal control. **c** Western blotting analysis for protein levels of MyoD by Flag antibody for 48 h in GM. **d** qPCR analysis was performed to detect the level of *ckm*、*cdh15* and *myh3* at 3d post-differentiation. **e** 293 T cells were transiently transfected with 4Rtk-luc and expression vector encoding the indicated proteins. Cells were harvested 48 h post-transfection. Firefly and Renilla luciferase activities were measured using the Dual-Luciferase Reporter Assay System Kit (Promega). The data was presented as the normalized ratio of Firefly luciferase activity to the Renilla luciferase activity. **f** The myotube formation was observed under white light after differentiation for 5 days. Scale bar =50 μm. **g** After transfection and differentiation for 6 days, MyHC was detected by immunofluorescence staining. Scale bar = 100 μm. Data are presented as mean ± S.E.M.; *n* = 3; *p < 0.05, ***p < 0.001 (Student’s t-test)

Six SNPs in *myoD* coding region (CDS) were discovered through whole-genome resequencing (Table 2). Among them, four SNPs do not result in amino acids alterations, whereas the other two SNPs at locus 41,424,009 (A → G) and 41,424,010 (C → G) synchronously encoded an amino acid, which resulted in the substitution of amino acid from arginine (Arg) to proline (Pro) at 76th amino acid of MyoD protein sequence (Fig. 2a). Of note, the substitution is mainly led by the alteration at locus 41,424,010 from C to G, which was appeared only in the lean pigs.

**Table 2:**
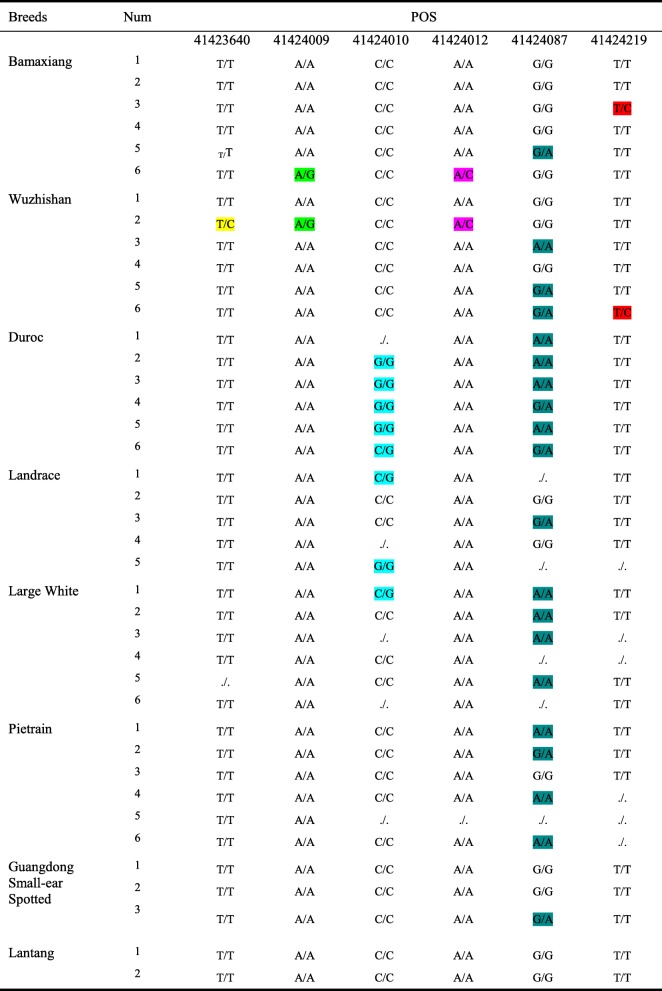
The SNPs in *myoD* CDS

(1) The SNPs in *myoD* CDS are shown and variations in each locus are represented by the colors indicated in the table

(2) “./.” represents that the site has not been detected

To determine whether this mutation can affect MyoD expression and then result in distinction in muscle mass, we constructed a *myoD* expressing plasmid with a C-terminal FLAG tag (PCDNA3.1-flag-*MyoD*^WT^) and a mutagenized plasmid to substitute Arg76 with Pro (PCDNA3.1-flag -*MyoD*^*R76P*^). These plasmids were separately transfected into NIH3T3 cells which cannot differentiate into myotubes because of lacking MyoD expression. When MyoD is forced to be expressed, NIH3T3 cell can differentiate into myotubes. So, it is considered as an ideal cell model for testing exogenous MyoD function. As a result, MyoD expression was up-regulated significantly both at mRNA level and protein level (Fig. 2b, c), which remarkably increased the mRNA levels of *Ckm, Cdh15 and Myh3* (Fig. 2d). Actually, *Ckm, Cdh15* and *Myog* are the targets of MyoD and promote myogenic differentiation. However, there was no significant difference between *MyoD*^*WT*^ and *MyoD*^*R76P*^ plasmid in the expression levels of *Ckm, Cdh15 and Myh3* (Fig. 2d). *MyoD*^*WT*^ and *MyoD*^*R76P*^ plasmids were respectively co-transfected into 293 T cells with a 4Rtk-luc reporter plasmid which was used as MyoD-responsive reporter [17] (Fig. 2e). The transfection of *MyoD*^WT^ and *MyoD*^R76P^ both could activate 4Rtk-luc transcription whereas there was no difference between them (Fig. 2e). Immunofluorescence assay showed that overexpression of *MyoD*^WT^ or *MyoD*^R76P^ in NIH3T3 cells equally promoted MyHC expression and generated myotubes (Fig. 2f, g). Collectively, these data made it clear that the mutant protein has neither effect on MyoD transcriptional activity in vitro, nor effect on myogenesis.

### MyoD clones across from amphibious to mammals can promote myogenesis

Based on the above-mentioned studies, we demonstrated that these SNPs in CDS and regulatory regions of *myoD* do not result in alteration of its expression and function. It should be noted that none of these mutations occurred in bHLH domain which presented high conservation among these pig populations. This phenomenon makes us have to believe that the bHLH domain of MyoD is conserved in function among normally growing animals, even different species.

**Fig. 3:**
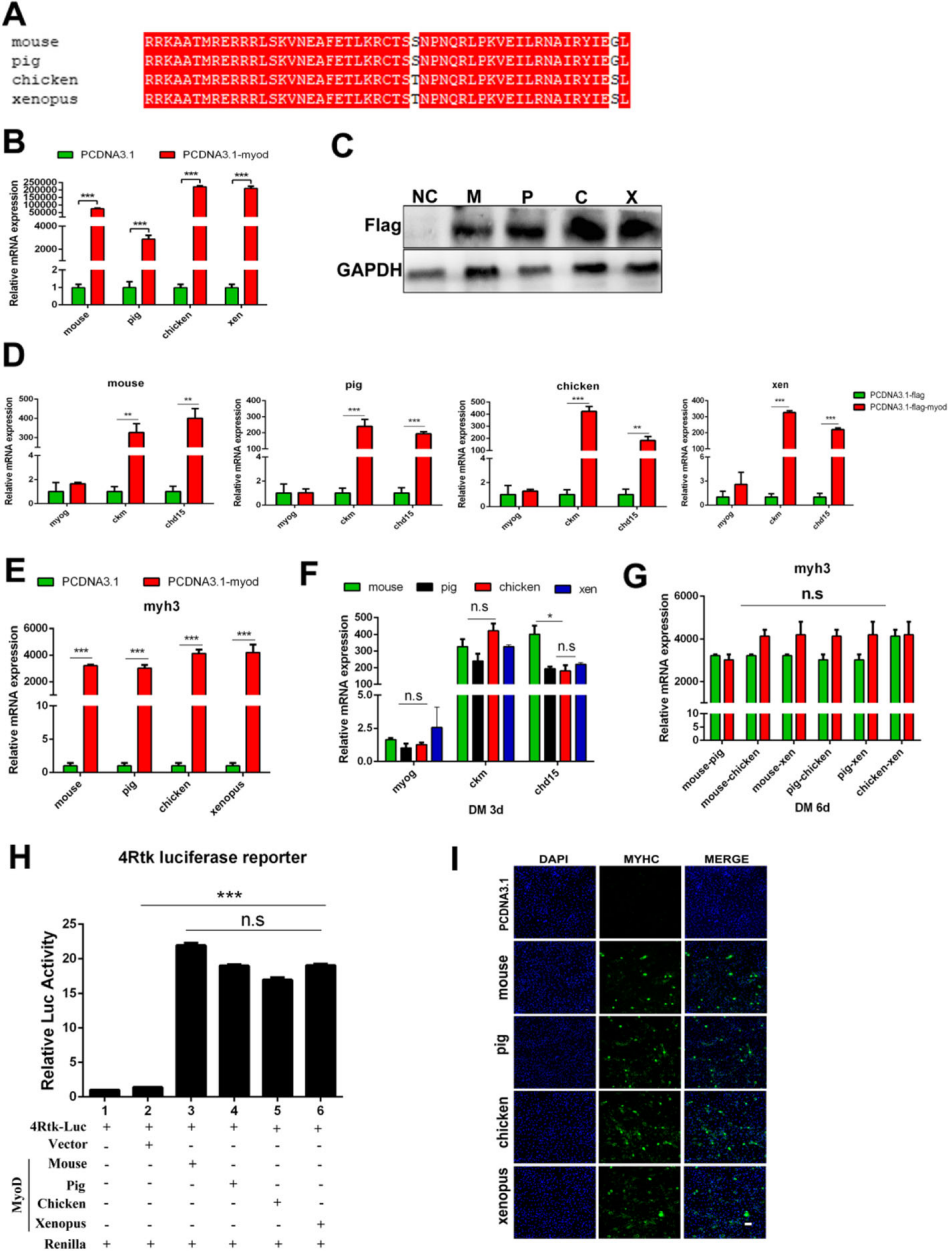
There is no difference in the transcriptional activity of MyoD among four species. **a** Alignment of bHLH domain among mouse, pig, chicken and xenopus. **b** qPCR analysis for the expression of *myoD* in NIH3T3 cells treated with overexpression Flag-*myoD* plasmid of different species or a control Flag vector in GM for 36 h. **c** After transfection of 48 h, MyoD protein level was detected by Flag antibody. Ctrl represents a control group. (**d, e**) qPCR analysis was performed to detect the expression level of *myog, ckm, cdh15* and *myh3* in different species at 3d (**d**) or 6d (**e**) post-differentiation. **f, g** The significance analysis of expression levels of key factors of myogenic differentiation from D and E in different species to determine whether MyoD has the same transcriptional activity. **h** 293 T cells were transiently transfected with 4Rtk-luc and *myoD* expression vectors of different species. After transfection of 48 h, cells were harvested to measure Firefly and Renilla luciferase activities by Dual-Luciferase Reporter Assay System Kit (Promega). **i** Immunofluorescence detection of MyHC (green) in *myoD* plasmid of mouse, pig, chicken and xenopus or control transfected NIH3T3 cells after 7 days of differentiation. Data are presented as mean ± S.E.M.; *n* = 3; *p < 0.05, **p < 0.01, ***p < 0.001 (Student’s t-test); Scale bar =100 μm

To validate the hypothesis, FLAG-tagged *myoD* plasmids were constructed using pig, mouse, chicken and xenopus tropicalis, respectively. The sequence homologies of MyoD protein among these four species vary from 66 to 88% (Table 3, Additional file 5: Figure S1.), whereas their bHLH regions are highly-conserved (98.08% identity) (Fig. 3a). When these *myoD* plasmids were transfected into NIH3T3 cells, the mRNA levels of *Ckm*, *Cdh15* and *Myh3* were all significantly increased (Fig. 3d, e). However, from the result, it was indicated that there was no significant disparity among the four species at the expression levels of *Ckm,* and *Myh3* regulated by MyoD (Fig. 3f, g). However, the expression level of *Cdh15* in mice was significantly higher than that in other three species (Fig. 3f), Which may be related to the fact that NIH3T3 is a mouse-derived cells. In order to assess the transcriptional activity of MyoD from different species, *myoD* plasmids was co-transfected into 293 T cells with 4Rtk-luc reporter plasmid. As expected, there is no difference of luciferase activity among them (Fig. 3h). Immunofluorescence assay also showed that overexpression of *myoD* clones promoted MyHC expression and resulted in the similar phenotype in NIH3T3 cells (Fig. 3i).

**Table 3:** The sequence homologies of MyoD among four species

Nucleotide / Protein BLAST						
Blast	mouse-pig	mouse-chicken	mouse-xenopus	pig-chicken	pig-xenopus	chicken-xenopus
Identities (%)	84.27	73.98	68.25	73.88	68.09	75.15
	88.09	65.62	66.25	65.72	65.73	75.58

(1) The sequence similarity comparison of MyoD gene and protein among different species

Together, these results indicated that all *myoD* clones across from amphibious to mammals can promote myogenesis of NIH3T3 fibroblasts cells owing to the highly-conserved bHLH domain, even though the rest amino acid residues are different.

## Discussion

The ongoing studies about swine have received extensive attention owning to its association to agricultural, clinical and dietary needs. Also, as one of the most significant livestock worldwide, pigs have been classified into lean, fat and miniature categories according to the differences in muscle and fat proportion, and it is well known that there are genetic differences among these three types of pig breeds. Lean pigs, such as Landrace, Duroc and Pietrain, are characterized by high lean meat percentage and fast-growing muscle [18–20]. In contrast, fat pigs including Lantang, Luchuan and Laiwu Blacks, as China indigenous pig breeds, are recognized by high subcutaneous fat content, slow-growing muscle as well as low lean percentage [18, 21]. As miniature pigs, Bamaxiang and Wuzhishan are not only relative slow in growth rate and feed conversion rate, but also less in muscle mass [22]. However, the genetic mechanism underlying the difference of muscle mass has remained unclear.

### The distinct muscle mass is not relevant to the SNPs in *myoD* gene

All SNPs in regulatory regions of *myoD* gene from 10 pig populations were listed in Tables 1 and 2. After analysis, it was inferred that the initiate up-regulated time and level of *myoD* expression should be not affected by the SNPs of *myoD* gene. The initiate expression of *myoD* occurred in myotome in mice and had crucial role in the specification of progenitor cells [3, 23]. Myotube formation was delayed in MyoD-deficient skeletal muscle [1, 24]. In particular, loss of MyoD facilitated adipogenic trans-differentiation of myoblasts [25]. So importantly *myoD* acts, it is not workable to expect *myoD* SNPs cause the alteration of *myoD* expression characteristics in normal muscle developing individuals. The mutations that affect muscle mass should generally exist in diseased individuals or individuals with phenotypic abnormalities. Although a novel variation of amino acid (Arg76Pro) was found in MyoD CDS, and only appeared in lean pigs (Table 2), this mutation did not alter the characteristics of *myoD* expression (Fig. 2a). Besides, this mutation also occurred in different species including pig, mouse, chicken and xenopus tropicalis (Additional file 5: Figure S1) whose MyoD transcriptional activity were similar in our experiment (Fig. 3). Therefore, this mutation (Arg76Pro) does not affect the transcriptional activity of MyoD, which was verified in NIH3T3 cells (Fig. 2). These data explained detailly that the distinct muscle mass among pig breeds is not relevant to the *myoD* SNPs which present in different pig populations with normal muscle development.

### No mutation occurs in the bHLH domain of MyoD in animals with normal muscle development

MyoD protein contains a bHLH motif, which can form dimers with the E-protein to promote MyoD transcriptional activity [26]. Previous studies have revealed that the bHLH domain of MyoD is sufficient to convert C3H10T1/2 cells to myoblasts [27]. What’s more, when the bHLH domain of MyoD is replaced by the bHLH domain of NeuroD2, MyoD will become a vital monitor of neurogenesis, other than a master regulator of myogenesis [28]. These results suggest that the bHLH domain of MyoD confers its own lineage determination potential. In addition, Myf5, a bHLH protein highly related with MyoD, is also expressed in skeletal muscle and has a crucial role in muscle cell specification. The mutation (p.Arg95Cys) located at the bHLH domain of Myf5 can impair Myf5 nuclear localization and transcriptional activity, which leads to external ophthalmoplegia, rib, and vertebral anomalies in humans [29]. Notably, the bHLH domain of MyoD is highly-conserved among pig breeds, even species in our study, indicating its importance in transcriptional activation. Since this domain is very important in myogenesis, it is unrealistic to expect functional mutations in this domain for normally developing individuals. In addition, it also indicates that difference in muscle mass among breeds is not triggered by the SNPs in bHLH domain, which was confirmed by our sequencing results.

### The prospect for exploring the mechanisms of different muscle mass

The difference in muscle mass among pig breeds is not due to the SNPs in *myoD* gene, which makes us more strongly believe that it is futile to explore the causes of the differences in muscle mass only based on SNPs in populations with normal muscle development. In general, if there is such a mutation that affects muscle mass, there will be abnormal muscle development, such as double muscle rump. After exclusion of SNPs at DNA level, the probable reason resulting in the difference of muscle mass could be attributed to epigenetic modifications that can also be inherited [9, 30, 31]. These modifications may affect the expression of MyoD by affecting its upstream regulatory factors and then influence the differentiation time of myoblasts, eventually leading to differences in muscle mass.

Overall, our study excludes a SNP-based strategy for exploring the mechanisms of different muscle mass among pigs in *myoD* or other important myogenic genes. At the same time, it also opens another door for researchers.

## Conclusion

In summary, our study has showed that the distinction of muscle mass among pig breeds is not caused by the SNPs in *myoD* gene, but might be attributed by epigenetic modifications, which conduce to understand the mechanisms underlying muscle mass among different pig breeds.

## Methods

### Sample collection and preparation

The pig populations used for SNPs in *myoD* gene were Bamaxiang (6, miniature), Wuzhishan (6, miniature), Duroc (6, lean), Landrace (5, lean), Large white (6, lean), Pietrain (6, lean), Guangdong Small-ear Spotted (3, fat), Lantang (3, fat), Luchuan (6, fat) and Laiwu Black (6, fat) pigs. The porcine ear tissues were snap-frozen in liquid nitrogen and stored until further use.

### DNA library construction and whole-genome resequencing

Porcine genome DNA was obtained using standard phenol chloroform method. One DNA library was constructed for each sample (Guangdong Small-ear Spotted and Lantang pigs) and then sequenced on an Illumina HiSeq X Ten system by Beijing Novogene (commercial service). We also downloaded other whole genome resequencing data of Bamaxiang, Wuzhishan, Duroc, Landrace, Large white, Pietrain, Luchuan and Laiwu Black pigs from GenBank. All the information of these samples was listed in Additional file 1: Table S1. Sequencing reads were mapped with porcine *myoD* in Sscrofa11.1 version by BWA-MEM [32] with default parameters and the polymorphisms of *myoD* were analyzed by GATK3.7 [33]. The sequencing depth of whole genome resequencing for each pig breed was listed in Table 4.

**Table 4:** The average depth of all samples

Breed	Depth range of sequencing (GB)
Bamaxiang	27.03 ~ 29.63
Wuzhishan	26.95 ~ 28.29
Duroc	8.90 ~ 17.68
Landrace	6.14 ~ 11.42
Large White	7.15 ~ 9.59
Pietrain	8.82 ~ 16.79
Guangdong Small-ear Spotted	39.18 ~ 41.24
Lantang	35.45 ~ 40.46
Luchuan	24.80 ~ 29.59
Laiwu Black	25.04 ~ 28.86

### Cell culture and differentiation

Mouse NIH3T3 cell line was purchased from ATCC, and were cultured in high-glucose DMEM with 10% fetal bovine serum (growth medium, GM) until confluence. NIH3T3 cells were switched into DMEM with 2% horse serum (differentiation medium, DM) when cells reached confluence. All cells were cultured at 37 °C in 5% CO2 incubator.

### Plasmids and transfection

The cDNA of *myoD* from mice, pigs, chicken and xenopus tropicalis were separately cloned into pcDNA3.1-flag vector (Invitrogen, Shanghai, China). NIH3T3 cells were transfected with *myoD* plasmid or control vector using Lipofectamine 3000 (Invitrogen) according to the manufacturer’s instruction. NIH3T3 cells were passaged to 6-well or 12-well plates 12 h before transfection. All transfections were performed in triplicate for each experiment.

### RNA isolation and quantitative real-time PCR

Total RNA was isolated from NIH3T3 cells using TRIzol® Reagent (Invitrogen, Shanghai, China) and cDNA was synthesized from total RNA by Reverse Transcription Kit (Promega, Shanghai, China). Quantitative real-time PCR (qPCR) assays was performed on LightCycler 480 II system (Roche, Basel, Switzerland) by using a SYBR Green qPCR Kit (Genestar, Beijing, China). *GAPDH* is used as an internal control for normalization. The sequences of qPCR primers are shown in Additional file 2: Table S2. The experimental data were analyzed using 2^-∆∆CT^ method.

### Western blotting

NIH3T3 cells were treated with cell Lysis Buffer to completely release total protein. Cell extracts were separated by SDS-PAGE and the proteins were transferred to 0.45 μm PVDF membrane (Bio-Rad, Shanghai, China). Then the PVDF membranes were blocked with 3% BSA for 1 h at room temperature and then incubated at 4 °C overnight with primary antibodies. After washing, the membranes were labeled with proper secondary antibodies. Blots were visualized using a commercial enhanced chemiluminescene detection kit (Thermo Scientific, Beijing, China). GAPDH was used as the internal control. Primary antibodies used in this study included Flag (#8146S, CST) and GAPDH (#AP0063, Bioworld). Secondary antibodies included either anti-rabbit HRP-linked (#7074 S, CST) or anti-mouse HRP-linked (#7076 S, CST) antibodies.

### Luciferase reporter assays

293 T Cells were seeded into a 24-well plate well and each plate of cells was transfected with 100 ng of 4R-TK-Luc and 100 ng of MyoD expression vectors or control vectors. Reporter activity was measured at 48 h later with a dual luciferase assay kit (Promega) and BioTek Synergy2. The luciferase activity was normalized by Renilla activity and the total transfection amount was normalized by an empty expression vector.

### Immunofluorescence assay

For myosin heavy-chain staining, cells in culture medium were rinsed with PBS, fixed with 4% paraformaldehyde, permeabilized with 0.5% Triton X-100 and blocked for 1 h with 3% BSA. Then incubated at 4 °C overnight with the anti-MyHC antibody (#ab51263, Abcam), washed three times with PBS and incubated for 1 h with secondary antibodies (#8940S, CST). After three washes by PBS, cells were stained with DAPI for 2 min. Images were captured by fluorescent reverse microscopy (ZEISS, Heidenheim, Germany).

### Statistical analysis

Data are presented as mean ± SE from three independent experiments. Statistical significance was determined by the Student’s t-test, and *P* < 0.05 was considered as significance (*P < 0.05; **P < 0.01; ***P < 0.001).

## Additional files

Additional file 1: **Table S1.** The information of all samples. (XLSX 295 kb)

Additional file 2: **Table S2.** The primer sequences for q-PCR. (DOCX 13 kb)

Additional file 3: **Table S3.** Summary of sequence similarity of *myoD* regulatory regions. (DOCX 15 kb)

Additional file 4: **Table S4.** Sequence size and location of *myoD* regulatory regions. (DOCX 13 kb)

Additional file 5: **Figure S1.** The similarity comparison of MyoD protein among different species. (DOCX 144 kb)

## Data Availability

The datasets supporting the results of this article are included within the article (and its additional files). The *myoD* sequence data of Small-ear Spotted and Lantang pigs have been deposited in GenBank database (Accession number: PRJNA530874).

## Abbreviations

Arg: Arginine
bHLH domain: Basic helix-loop-helix domain
CDS: Coding region
CE: Core enhancer
DM: Differentiation medium
DRR: Distal regulatory region
GM: Growth medium
MyoD: Myogenic Differentiation 1
Pro: Proline
PRR: Proximal regulatory region
qPCR: Quantitative real-time PCR
SNPs: Single nucleotide polymorphisms
TSS: Transcriptional start site
