# Supplementary material for: The SNPs in myoD gene from normal muscle developing individuals have no effect on muscle mass
Source: BMC Genet. 2019 Sep 2;20:72. doi: 10.1186/s12863-019-0772-6 (PMC6720383; doi:10.1186/s12863-019-0772-6)
Supplement: Supplementary file 2 — Table S2. The primer sequences for q-PCR. (DOCX 13 kb) [file 12863_2019_772_MOESM2_ESM.docx]

**Table S2.** The primer sequences for q-PCR

| Gene | Sequences | |
| --- | --- | --- |
| MyoD(mouse) | F: CCACTCCGGGACATAGACTTG | R: AAAAGCGCAGGTCTGGTGAG |
| MyoD (pig) | F: CAGCGGACGACTTCTATGATGACCC | R: GCGGCAGGGAAGTGCGAGTGTT |
| MyoD (chicken) | F: CGGCTCAGCAAGGTCAACGAG | R: GAATCTGGGCTCCACTGTCACTC |
| MyoD (xenopus) | F: CCCGATGACTTCTACGACG | R: GGTCCAGGTCCTCAAAGAA |
| Myog | F: GAGACATCCCCCTATTTCTACCA | R: GCTCAGTCCGCTCATAGCC |
| Ckm | F: CTGACCCCTGACCTCTACAAT | R: CATGGCGGTCCTGGATGAT |
| Cdh15 | F: CATCCCACCCATTAGTGTGTC | R: CCTCCCGGTGAACTTGTCG |
| Myh3 | F: AAAAGGCCATCACTGACGC | R: CAGCTCTCTGATCCGTGTCTC |
| GAPDH | F: AGGTCGGTGTGAACGGATTTG | R: TGTAGACCATGTAGTTGAGGTCA |
