# Supplementary material for: The SNPs in myoD gene from normal muscle developing individuals have no effect on muscle mass
Source: BMC Genet. 2019 Sep 2;20:72. doi: 10.1186/s12863-019-0772-6 (PMC6720383; doi:10.1186/s12863-019-0772-6)
Supplement: Supplementary file 3 — Table S3. Summary of sequence similarity of myoD regulatory regions. (DOCX 15 kb) [file 12863_2019_772_MOESM3_ESM.docx]

**Table S3.** Summary of sequence similarity of *MyoD* regulatory regions

| Nucleotide BLAST | | | | | | | | | | | |
| --- | --- | --- | --- | --- | --- | --- | --- | --- | --- | --- | --- |
|  |  | CE |  |  |  | DRR |  |  |  | PRR |  |
| Blast | human-mouse | human-pig | mouse- pig |  | human-mouse | human-pig | mouse- pig |  | human-mouse | human-pig | mouse- pig |
| Identities (%) | 89 | 94 | 92 |  | 71 | 79 | 68 |  | 70 | 76 | 71 |

Sequence similarity comparison using DNAMAN software
