# Supplementary material for: The SNPs in myoD gene from normal muscle developing individuals have no effect on muscle mass
Source: BMC Genet. 2019 Sep 2;20:72. doi: 10.1186/s12863-019-0772-6 (PMC6720383; doi:10.1186/s12863-019-0772-6)
Supplement: Supplementary file 4 — Table S4. Sequence size and location of myoD regulatory regions. (DOCX 13 kb) [file 12863_2019_772_MOESM4_ESM.docx]

**Table S4.** Sequence size and location of *MyoD* regulatory regions

| Statistics of fundamental feature | | | | | | | | | | | |  |
| --- | --- | --- | --- | --- | --- | --- | --- | --- | --- | --- | --- | --- |
|  | CE | | |  | DRR | | |  | PRR | | | |
| Species | human | mouse | pig |  | human | mouse | pig |  | human | mouse | pig | |
| Length（bp） | 258 | 258 | 258 |  | 795 | 721 | 728 |  | 302 | 300 | 289 | |
| Distance from TSS (kb) | **-22~**  **-18** | **-22~**  **-18** | **-22182bp**  **~**  **-21924bp** |  | **-5.4~**  **-4.7** | **-5.4~**  **-4.7** | **-5.4~**  **-4.7** |  | **-275bp**  **~20bp** | **-275bp**  **~20bp** | **-485bp**  **~ -197bp** | |
