# Supplementary figures and images for: The SNPs in myoD gene from normal muscle developing individuals have no effect on muscle mass
Source: BMC Genet. 2019 Sep 2;20:72. doi: 10.1186/s12863-019-0772-6 (PMC6720383; doi:10.1186/s12863-019-0772-6)

**
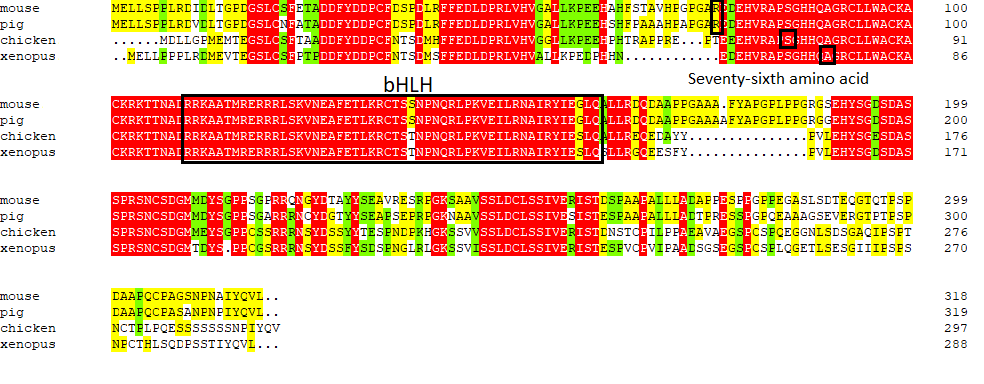
**

**Figure S1.** The similarity comparison of MyoD protein among different species

Supplement: Supplementary file 5 — Figure S1. The similarity comparison of MyoD protein among different species. (DOCX 144 kb) [file 12863_2019_772_MOESM5_ESM.docx]
